# Automatic Assignment of EC Numbers

**DOI:** 10.1371/journal.pcbi.1000661

**Published:** 2010-01-29

**Authors:** Volker Egelhofer, Ida Schomburg, Dietmar Schomburg

**Affiliations:** Department of Bioinformatics and Biochemistry, Technical University Braunschweig, Braunschweig, Germany; University of Washington, United States of America

## Abstract

A wide range of research areas in molecular biology and medical biochemistry require a reliable enzyme classification system, e.g., drug design, metabolic network reconstruction and system biology. When research scientists in the above mentioned areas wish to unambiguously refer to an enzyme and its function, the EC number introduced by the Nomenclature Committee of the International Union of Biochemistry and Molecular Biology (IUBMB) is used. However, each and every one of these applications is critically dependent upon the consistency and reliability of the underlying data for success. We have developed tools for the validation of the EC number classification scheme. In this paper, we present validated data of 3788 enzymatic reactions including 229 sub-subclasses of the EC classification system. Over 80% agreement was found between our assignment and the EC classification. For 61 (i.e., only 2.5%) reactions we found that their assignment was inconsistent with the rules of the nomenclature committee; they have to be transferred to other sub-subclasses. We demonstrate that our validation results can be used to initiate corrections and improvements to the EC number classification scheme.

## Author Summary

The fundamental understanding of metabolism in organisms which can only be achieved by integrated studies on their biology using a systems biology approach will aid in the design of future metabolic engineering strategies. Metabolic network reconstruction provides insight into the molecular mechanisms of a particular organism. An annotated genome containing the specific metabolic genes found in a particular organism can be used to reconstruct its metabolic network. The correlation between the genome and metabolism is made by searching gene databases or by searching protein databases with a known EC number in order to find the associated gene. The success of the search process is critically dependent upon the consistency and reliability of the underlying data. Therefore we have developed tools which can be used to identify wrong or inconsistent classification of enzymes and help to remove them from the relevant search databases.

## Introduction

**Figure 1:**
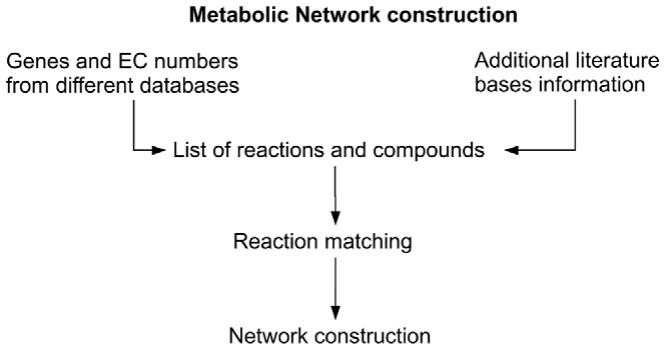
Processes for reconstruction of a metabolic network.

With the several thousand proteins found in each organism a highly developed hierarchical and consistent classification scheme is absolutely essential for a comparison of metabolic capacities of the organisms. Unfortunately such a system exists only for the enzymes and not for the other protein classes but for the enzymes the classification scheme allows an immediate access or the enzyme functional properties including catalysed reaction, substrate specificity, etc. In this respect a quick comparative assessment of enzymatic pathways between organisms is possible even when the enzymes in the different organisms have totally different sequences as long as they belong to the same EC-class. A well reconstructed metabolic network provides a unified platform to integrate all the biological and medical information on genes, enzymes, metabolites, drugs and drug targets for a system level study of the relationship between metabolism and disease. Therefore an accurate representation of biochemical and metabolic networks by mathematical models is one of the major goals of integrative systems biology. Metabolic networks have been constructed for a number of genomes [1],[2]. An example for the reconstruction process of a metabolic network are schematically shown in Figure 1. It is essential to integrate information from different databases to get a more complete enzyme list for the reconstruction. The main databases to be taken into account to provide a complete cross-link between genes and their corresponding enzymes are NCBI EntrezGene [3], Ensembl [4], KEGG [5], MetaCyc [6] and BRENDA [7]. The second step of the reconstruction procedure is to fill the gaps resulting from the first step based on information from literature. This step is very time-consuming and it would be therefore highly desirable to make the first step an automatic and reliable procedure. One of the problems is the different substrate specificity of enzymes in different organisms a fact that cannot be really accounted for by any classification system [8]. A further problem is the wide-spread use of incomplete EC numbers such as 1.-.-.- (e.g. in UNIPROT entry AK1C3_HUMAN). This often occurs because an enzymatic function is inferred from the existence of a certain pair of metabolites or only experimentally shown from a cell extract without a full characterisation of the enzyme with biochemical methods, which is the requirement for the assignment of EC-numbers by the IUBMB Nomenclature Committee [9]. For example, in the UniProt database there are more than 800 proteins annotated with an incomplete EC number [10]. Applications like drug design, ligand docking, or systems biology require the EC number classification to be correct, consistent, and accurate. For these reasons the automatic assignment of EC numbers to enzymatic reactions is a current issue in bioinformatics and requires specific chemical knowledge, therefore just a few approaches have been published to handle the assignment problem. The Kyoto Encyclopedia of Genes and Genomes (KEGG) developed a tool for computational assignment of EC numbers published by Kotera et al. [11]. In this approach each reaction formula is decomposed by manual work into sets of corresponding substrate and product molecules, which are called reactant pairs. In the second step every reactant pair is analysed by the structure comparison method SIMCOMP developed by Hattori et al. [12]. Another approach proposed by Körner et al. [13] and Apostolakis et al. [14] considers reaction energetics to predict reaction sites. Lationa et al. [15] introduced an EC number classification method based on self-organizing maps. This approach allows to assign EC numbers at the sub-subclass levels for reactions with accuracies of 70%. One of the authors being the current chairman of the IUBMB nomenclature committee we felt the need to develop a system that allows for a highly reliable classification system that can help to identify the sub-subclass of any given enzyme-catalyzed reaction, allow a quick assignment of new reactions and additionally serve in a retrospective quality control of existing EC-numbers. With ca. 4000 existing EC-numbers this can certainly not be done by hand. In this article we present an efficient and reliable strategy for the automatic classification of enzyme-catalysed biochemical reactions based on the chemical structure of the involved substrates and products.

## Results/Discussion

The objective of the study was the automatic assignment of reactions to the EC number classification system. The approach is designed to adapt the EC number classification system as closely as possible. Therefore in most cases the results corresponds to the given sub-subclass by the IUBMB, but it some cases it differs from the established classification. We decided to subdivide the results into nine different subsets.

**Figure 2:**
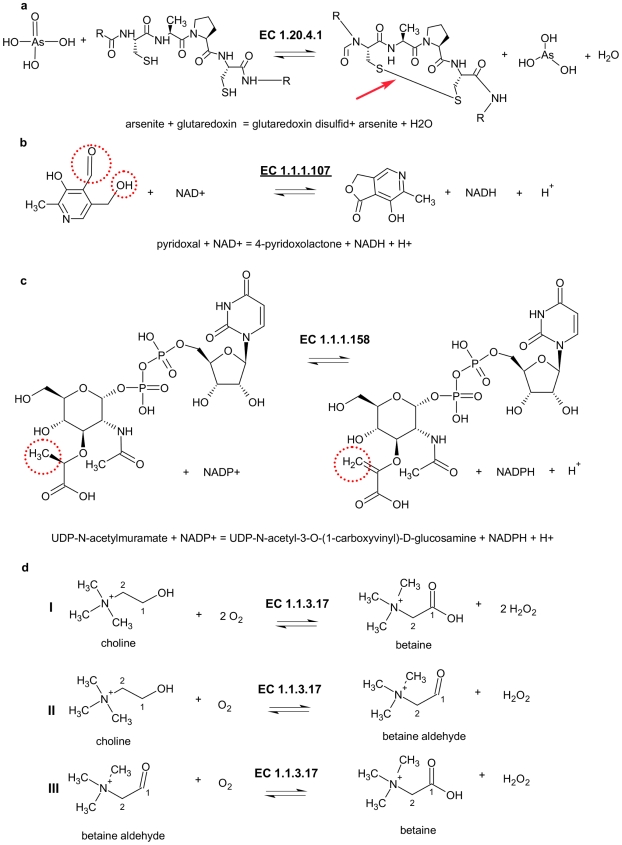
Examples for the different subsets. (a) The reverse direction of the reaction is shown. (b) Ambiguous, fits more than one sub-subclass. (c) Reaction is assigned to a wrong sub-subclass. (d) The enzyme catalysis two or more different types of reaction, where at least one does not meet the requirements of the assigned sub-subclass.

As shown in Table 1, subset 2 covers all reactions in the EC system where instead of the correct – the reverse direction of reaction is shown. For example the reaction catalysed by arsenate reductase (EC 1.20.4.1, see Figure 2a) assigned to the sub-subclass 1.20.4 which covers enzymes ‘Acting on phosphorus or arsenic in donors, with a disulfide as acceptor’ as defined by the NC-IUBMB.

**Table 1:** The dataset consists of 3,788 enzyme-catalysed biochemical reactions.

subset	NO. of reactions	description
1	3115	agreement of our assignment with EC-classification
2	12	reverse direction of reaction was listed
3	86	ambiguous, fits more than one sub-subclass
4	61	Reaction assigned to a wrong sub-subclass
5	18	catalysis of two or more different types of reaction, where at least one does not meet the requirements of the assigned sub-subclass.
6	92	unclear assignment
7	17	ambiguous, fits two or more quite similar sub-subclasses
8	9	Does not fit any defined sub-subclass
9	378	different sub-subclasses assigned, based on the identical reaction

We predict nine different case groups.

A reaction catalysed by pyridoxal 4-dehydrogenase represents an example of subset 3 (Figure 2b). This enzyme had been assigned the sub-subclass 1.1.1 which includes enzymes ‘Acting on the CH-OH group of donors, with NAD^+^ or NADP^+^ as acceptor’, but it can also be assigned the sub-subclass 1.2.1 which covers enzymes ‘Acting on the aldehyde or oxo-group of donors, with NAD^+^ or NADP^+^ as acceptor’.

Subset 4 contains enzymes where the assignment is definitely inconsistent assigned with the NC-IUBMB rules (Table S1). For example the reaction catalysed by UDP-N-acetylmuramate dehydrogenase with EC Number 1.1.1.158 (see Figure 2c) is identified by our approach as an enzyme acting on the CH-CH group of donors, with NAD^+^ or NADP^+^ as acceptor which corresponds to sub-subclass 1.3.1 as it is defined by the NC-IUBMB. The transfer of the EC Number of 1.1.1.158 into sub-subclass 1.3.1 issued on our initiative is already accepted by the IUBMB. The other 60 errors have also been reported to the IUBMB and are currently under examination.

Choline oxidase (EC 1.1.3.17) an example of subset 5 of our results is a bifunctional enzyme which catalyses two different kinds of reactions. The overall reaction shown is Figure 2d (Reaction I). On the one hand the enzyme is acting on the CH-OH group of the choline, with oxygen as acceptor (Figure 2d, Reaction II), which marks the enzyme as an oxireductase of sub-subclass 1.1.3, on the other hand the enzyme is acting on the aldehyde of betaine, with oxygen as acceptor (Figure 2d, reaction III) which is characteristic for an oxireductase of sub-subclass 1.2.3 as defined by the NC-IUBMB. In these cases two EC-numbers should be assigned to the enzyme.

**Figure 3:**
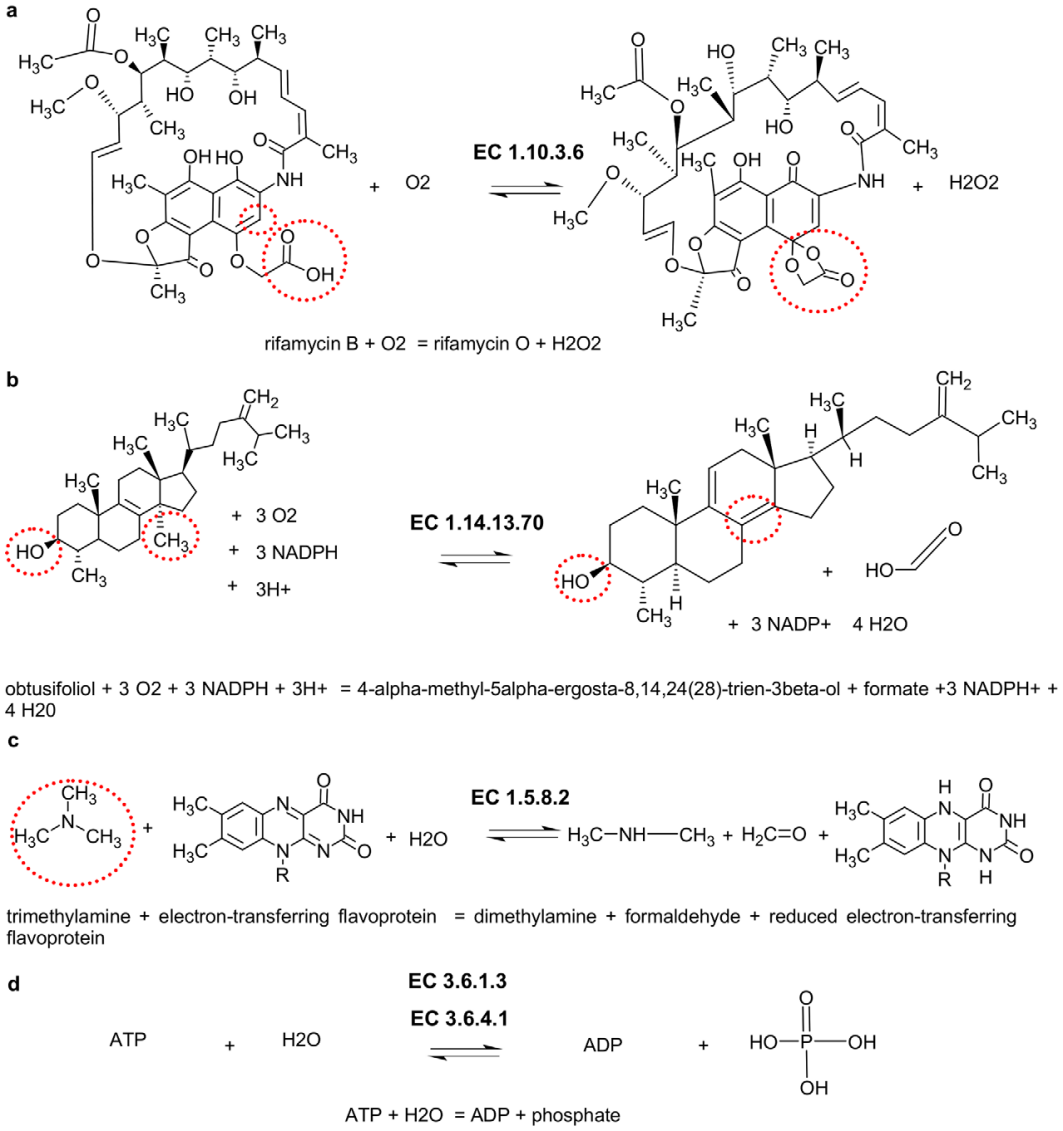
Examples for the different subsets. (a) Unclear assignment (b) Ambiguous, fits two or more quite similar sub-subclasses. (c) Does not fit any defined sub-subclass. (d) Different sub-subclasses assigned, based on the identical reaction.

The subset 6 involves all enzymes catalysing reactions which are identified as unclear assignement. The reaction shown in Figure 3a is assigned to sub-subclass 1.10.3 in which enzymes are classified acting on diphenols and related substances as donors, with an oxygen as acceptor. This usually includes the reduction of one or both hydroxyl groups of the involved phenol, but in this case a carboxyl group reacts with a carbon ring-atom and as a result another ring is formed.

An example for subset 7 is shown in Figure 3b. The reaction catalysed by the sterol 14-demethylase (1.14.13.70) is correctly assigned to sub-subclass 1.14.13 which compromise enzymes ‘acting on paired donors, with incorporation or reduction of molecular oxygen, with NADH or NADPH as one donor, and incorporation of one atom of oxygen’ but it also could be assigned to sub-subclass 1.14.21 which contains enzymes ‘acting on paired donors, with incorporation or reduction of molecular oxygen, with NADH or NADPH as one donor, and the other dehydrogenated’. These two sub-subclasses are too similar and therefore could easily be merged without loss of information.

Subset 8 is composed of enzymes which could not be clearly assigned to any defined sub-subclass. For example the trimethylamine dehydrogenase is assigned to sub-subclass 1.5.8 which contains enzymes ‘Acting on the CH-NH group of donors,with a flavin as acceptor’, but as shown in Figure 3c the substrate trimethylamine has no CH-NH group, the enzyme could be described as ‘Acting on other nitrogenous compounds as donors,with a flavin as acceptor’ but this sub-subclass (1.7.8) does not exist in the EC number classification scheme so far.

In subset 9 we have summarized enzymes where the assignment to a subclass is not unequivocally determined by the chemical reaction given. The reaction ATP + H2O = ADP + phosphate as shown in Figure 3d is catalysed by the enzymes adenosinetriphosphatase (3.6.1.3) and myosin ATPase (3.6.4.1). In the case of 3.6.4.1 the ATPase activity is connected to actin movement, and in 3.6.1.3 this is not so. Here the general principle that the enzyme class is defined by its chemical reaction is violated. The same is true for the peptidases (subclass 3.4) that are classified according to the mechanism, not the reaction [16].

Our approach has been used for the classification of 3788 enzymatic reactions including 229 sub-subclasses of the EC classification system. We demonstrated that enzyme-catalysed reactions can be classified efficiently and reliably by our approach. Furthermore, reactions can be assigned even if full characteristics of enzymes are not known. Moreover we have shown that this method can be used to identify wrong or inconsistent classification of enzymes and help to remove them.

### Conclusions

With one of the authors being the present chairman of the NC-IUBMB it is planned to use this and related tools to identify and remove errors and inconsistencies in the current EC-system and to optimise the system in a transparent and stable way. We plan to develop a tool that assign EC sub-subclasses to new reactions, access to which will be provided to the scientific community in the Internet’.

## Materials And Methods

### Data Sets

We used 3,788 different enzyme-catalysed reactions from an in-house-developed Database named BiReDa (Biochemical Reaction Database). The database held exclusively error-free MDL/MOL files as well as stoichiometrically and stereochemically correct reaction data from the BRENDA Database [7] and the KEGG LIGAND database [5], which have been corrected manually or automatically, if required.

### Procedure for automatic assignment of EC numbers

The key idea of this approach is to reproduce the classification system given by the IUBMB as closely as possible and not to create new classification rules. The underlying procedure is divided into two steps:

#### STEP 1

The chemical similarity calculation

##### 1.1 Coding of atoms

In order to identify the corresponding partners within a biochemical reaction every atom of each compound is coded as follows:

sCCCOOOHNNNSSSPPPRRRAsAsAsMMMXXXc

where ‘s’ is the symbol of the corresponding element of the given atom and each other letter represents the symbols of the connected atoms except for a few exceptions: ‘R’ stands for any rest, ‘M’ represents any metal ion, ‘X’ is any halogen and ‘c’ is the charge of the considered atom. In most cases there are three entries for each symbol: e.g. ‘CCC’, the first position represents the number of carbon atoms connected via a single bond, the second the number of atoms connected via a double bond and the third the number of atoms connected via a triple bond with the given atom. In the case of ‘H’ only one placeholder is needed because hydrogen forms only single bonds. A few examples of complete atom coding operators are shown in Table 2.

**Table 2:** Examples for the atom coding.

compound	sCCCOOOHNNNSSSPPPRRRAsAsAsMMMXXXc
CH_4_	C00000040000000000000000000000
H_2_C = O	C00001020000000000000000000000
^(+)^ NH_4_	N00000040000000000000000000001

##### 1.2 Coding of bonds

In addition to the atoms which are affected in the enzyme-catalyzed reactions, the bonds cleaved have to be identified. This in particular is necessary for the lyases which catalyzes the breakage of a carbon-oxygen, carbon-carbon or carbon-nitrogen bond in non-oxidative manner (e.g. enzymes assigned to sub-subclass 4.2.1 defined as enzymes which catalyse the breakage of a carbon-oxygen bond).

Therefore each bond is coded as follows:
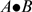
where ‘A’ is the first atom, ‘B’ is the second atom and ‘x’ is the bond type between these two atoms. For example a single carbon-carbon bond is coded as ‘C-C’, the code for a double carbon = carbon bond is ‘C = C’ and a nitrogen molecule is coded as ‘N#N’.

##### 1.3 Molecule similarity calculation

For the scoring scheme describing the similarity between each substrate and product molecule the Tanimoto Coefficient was used [17]:
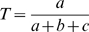
where:

‘a’ is the sum of the number of atom-types and bond-types which have the same frequency of occurrence in both the given substrate and the given product.

‘b’ is the number of atom-types and bond-types which have a higher frequency of occurrence in the given substrate than in the corresponding product molecule.

‘c’ is the number of atom-types and bond-types which have a lower frequency of occurrence in the given substrate than in the corresponding product molecule.

‘T’ is the Tanimoto coefficient which lies between 0 for unequal and 1 for identical molecules.

As a result we obtain a list of substrate/product pairs sorted according to their similarity.

#### STEP 2

The characterization of the individual reaction

##### 2.1 Identification of known reaction pairs

At the beginning compounds of known substrate/product pairs (see Table 3), which are part of many biochemical reactions are identified by the given InChIKey [18].

**Table 3:** Some examples of known substrate/product pairs.

substrate	product
NAD^+^	NADH
NADP^+^	NADPH
NADH	NAD^+^
NADPH	NADP^+^
H_2_O	NH_3_
OXOGLUTARATE	SUCCINATE
GLUTATHIONE DISULFIDE	GLUTATHIONE
O_2_	H_2_O_2_

##### 2.2 Coding of functional groups

In this step the atom coding operators generated during chemical similarity calculation (Step 1) are used to identify the important molecular functional groups responsible for the characteristics of each biochemical reaction.

As an example a carboxilic acid is shown in Table 4. The identification is done via two coding operators, one represents the C-atom and its environment (CO = O) and the other the directly connected O-atom of the hydroxyl group (OH-C).

**Table 4:** Examples for the functional group determination.

compound	code
carboxylic acid	CA
composition	atom coding operator
CO = O	C00011000000000000000000000000
OH-C	O10000010000000000000000000000

##### 2.3 Coding of molecule structure

In some cases it is necessary to identify known complex chemical structures such as ‘heme-groups’, ‘phenols’ or ‘iron-sulfur complexes’ etc. which represent only a part of a given molecule. In order to identify such complex structures it is unavoidable to identify also complex parts of a molecule like rings and ring-systems. For example a ‘heme-group’ is identified by its four pyrrole rings, connected via the central iron atom. Furthermore to distinguish between Fe^2+^ and Fe^3+^ the charge of the iron atom has to be taken into account too (Table 5).

**Table 5:** Examples for the molecular structure coding.

structure	code
cytochrome (oxidized)	CYO
cytochrome (reduced)	CYR
iron-sulfur (oxidized)	ISO
iron-sulfur (reduced)	ISR
flavin (oxidized)	FLO
flavin (reduced)	FLR

##### 2.4 Identification of unknown reaction pairs

**Figure 4:**
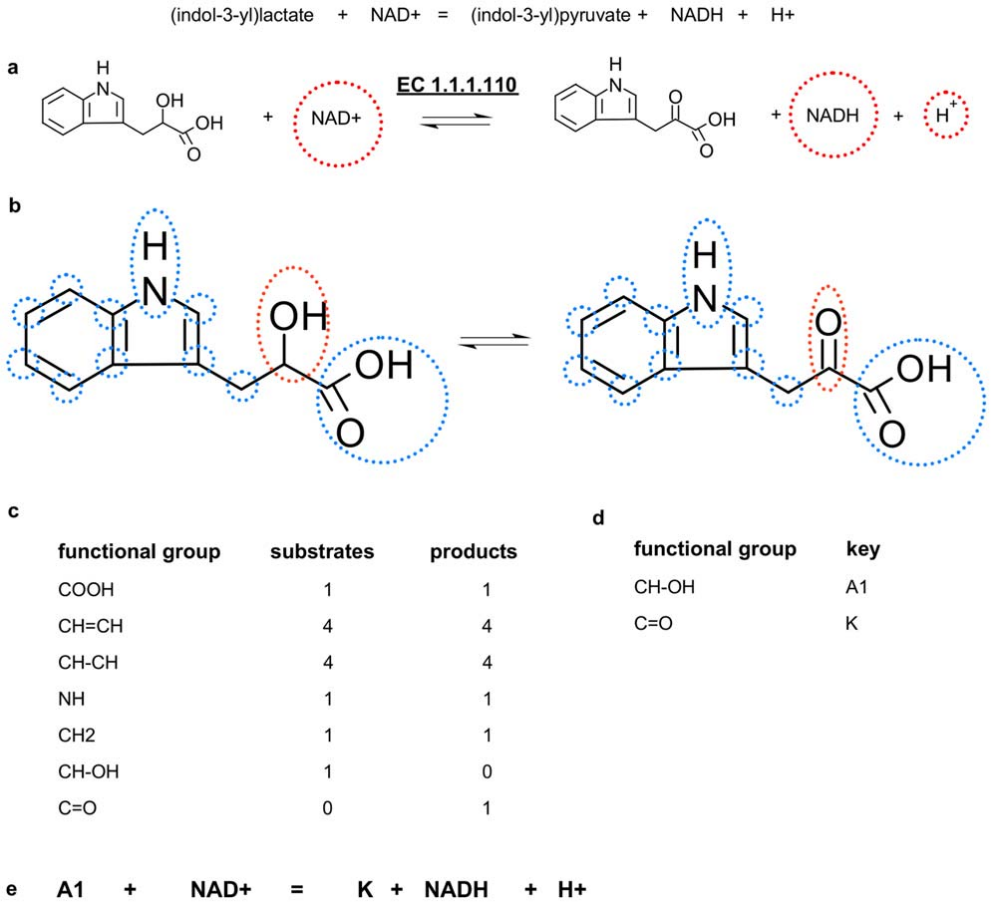
The complete procedure is demonstrated for the reaction catalysed by the enzyme indolelactate dehydrogenase (EC 1.1.1.110). (a) In the first step the known reaction pairs NAD^+^/NADH and accordingly NAD^+^/H^+^ are identfied and removed from further calculation steps. (b) The functional groups within the remaining molecules are identified (c), counted and eliminated in the case if they are equal in number. (d) For each remaining group a distinct key is assigned. (e) Finally, a difference key of the overall reaction is generated.

Starting with the most similar substrate/product pair in a given reaction the type and number of atoms, bonds, functional groups and structures between each substrate/product pair are compared and the differences are recorded in a new list. The outcome of each substrate/product comparison step is a difference key which is a string of all different types for a given substrate/product pair. The types which are identical are eliminated during each comparison step in order to prevent mismatches in the next turn. As an example the reaction catalysed by the enzyme indolelactate dehydrogenase (EC 1.1.1.110) is shown in Figure 2. In the first Step the known reaction pairs NAD^+^/NADH and accordingly NAD^+^/H^+^are identified and removed from further calculation steps (Figure 4a). As a result, the substrate (indol-3-yl)lactate and the product (indol-3-yl)pyruvate are left over. Now the functional groups within the molecules are identified (Figure 4b), counted (Figure 4c) and eleminated if they are equal in number. For each remaining group a distinct key is assigned (Figure 4d) and finally a difference key of the overall reaction is generated (Figure 4e). The above mentioned difference key of the overall reaction catalysed by EC 1.1.1.110 is defined by:
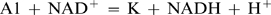
where are ‘A1’ is the code for a primary alcohol and ‘K’ represents a ketone group. This difference key represents enzymes which are part of EC-sub-subclass: ‘1.1.1’. We have defined at least one and if necessary more than one unique difference keys for each sub-subclass of the EC Number classification system.

## Supporting Information

Table S1 Wrong assigned EC Numbers and new suggested Sub-Sub-Classes. Contains enzymes which were definitely wrong assigned by the NC-IUBMB.
(0.02 MB DOC)
