# Supplementary material for: Automatic Assignment of EC Numbers
Source: PLoS Comput Biol. 2010 Jan 29;6(1):e1000661. doi: 10.1371/journal.pcbi.1000661 (PMC2813261; doi:10.1371/journal.pcbi.1000661)
Supplement: Table S1 — Wrong assigned EC Numbers and new suggested Sub-Sub-Classes. Contains enzymes which were definitely wrong assigned by the NC-IUBMB. (0.02 MB DOC) [file pcbi.1000661.s001.doc]

Table S1 Wrong assigned EC Numbers and new suggested sub-subclasses

| EC Number | suggested sub-subclass |
| --- | --- |
| 1.3.99.8 | 1.17.99 |
| 2.3.1.101 | 2.1.2 |
| 2.3.1.117 | 2.3.3 |
| 3.1.1.74 | 3.3.2 |
| 3.5.3.19 | 3.5.1 |
| 3.5.99.1 | 3.5.4 |
| 3.5.99.3 | 3.5.4 |
| 3.5.99.4 | 3.5.4 |
| 3.5.4.22 | 3.5.99 |
| 4.2.99.12 | 4.1.3 |
| 4.2.1.87 | 4.3.1 |
| 4.2.1.88 | 4.3.3 |
| 2.5.1.4 | 4.4.1 |
| 5.3.99.7 | 5.5.1 |
| 5.3.99.8 | 5.5.1 |
| 5.3.99.8 | 5.5.1 |
| 5.3.99.9 | 5.5.1 |
| 6.3.2.22 | 6.3.1 |
| 2.7.8.23 | 4.1.1 |
| 3.2.1.147 | 3.3.1 |
| 3.1.1.58 | 3.5.1 |
| 1.1.1.246 | 1.17.1 |
| 1.1.1.158 | 1.3.1 |
| 1.1.1.203 | 1.2.1 |
| 1.1.1.252 | 1.3.1 |
| 1.1.3.14 | 1.10.3 |
| 1.13.12.12 | 1.13.11 |
| 1.13.12.14 | 1.14.13 |
| 1.13.12.14 | 1.14.21 |
| 1.14.13.35 | 1.14.12 |
| 1.14.13.43 | 1.14.12 |
| 1.14.99.31 | 1.14.21 |
| 1.14.99.32 | 1.14.21 |
| 1.14.99.34 | 1.14.13 |
| 1.14.99.36 | 1.13.11 |
| 1.21.4.1 | 1.4.4 |
| 1.3.1.41 | 1.5.1 |
| 1.3.1.63 | 1.97.1 |
| 1.3.3.9 | 1.14.21 |
| 1.5.1.12 | 1.7.1 |
| 1.5.1.15 | 1.7.1 |
| 1.5.1.27 | 1.7.1 |
| 1.5.1.5 | 1.7.1 |
| 1.5.3.10 | 1.7.3 |
| 1.5.3.12 | 1.7.3 |
| 1.5.3.12 | 1.7.3 |
| 1.5.3.12 | 1.7.3 |
| 1.5.99.2 | 1.7.99 |
| 1.5.99.4 | 1.17.99 |
| 3.5.3.21 | 3.5.1 |
| 3.5.3.4 | 3.5.1 |
| 3.5.4.8 | 3.5.99 |
| 3.6.1.9 | 3.1.4 |
| 4.2.1.51 | 4.1.1 |
| 4.2.1.91 | 4.1.1 |
| 1.3.1.24 | 1.7.1 |
| 1.3.1.36 | 1.7.1 |
| 1.3.3.4 | 1.7.3 |
| 1.3.3.5 | 1.7.3 |
| 1.4.1.17 | 1.5.1 |
| 1.13.99.3 | 1.13.11 |
